# The role of the peripheral immune system in mediating axonal dysfunction in early-stage amyotrophic lateral sclerosis: An age- and sex-based analysis

**DOI:** 10.4103/NRR.NRR-D-24-01081

**Published:** 2025-03-25

**Authors:** Zhuoya Wang, Wen Cao, Lu Chen, Shuo Zhang, Lu Tang, Wenjuan Cui, Mingjun Kong, Ling Yu, Dongsheng Fan, Wei Zheng

**Affiliations:** 1 Department of Neurology, Peking University Third Hospital, Beijing, China; 2 Beijing Key Laboratory of Biomarker and Translational Research in Neurodegenerative Diseases, Beijing, China; 3 Key Laboratory for Neuroscience, National Health Commission/Ministry of Education, Peking University, Beijing, China

**Keywords:** amyotrophic lateral sclerosis, axonal degeneration, compound muscle action potential, disease progression, mediation analysis, neutrophil, neutrophil to lymphocyte ratio, peripheral immunity, systemic immune inflammation index, total leukocytes

## Abstract

Amyotrophic lateral sclerosis is characterized by the progressive loss of motor neurons. Early-stage axonal dysfunction, rather than central nervous system injury, plays a key role in the disease process. However, the molecular mechanisms underlying this dysfunction remain unclear. To investigate the relationship between peripheral immune dysregulation and axonal dysfunction in amyotrophic lateral sclerosis, we recruited 372 patients within the first 12 months of sporadic amyotrophic lateral sclerosis onset between January 2018 and May 2024. We collected peripheral immune markers at baseline, including total leukocytes, lymphocytes, monocytes, neutrophils, basophils, eosinophils, and platelets. We also calculated four derived ratios: neutrophil-to-lymphocyte ratio, platelet-to-lymphocyte ratio, lymphocyte-to-monocyte ratio, and systemic immune inflammation index. Multivariate analysis, adjusted for confounding factors, revealed that higher counts of total leukocytes and neutrophils, as well as higher neutrophil-related ratios, including the neutrophil to lymphocyte ratio and the systemic immune inflammation index, were significantly correlated with higher compound muscle action potential scores. Stratified analyses revealed that these associations varied by age and sex. Furthermore, mediation analysis demonstrated that axonal dysfunction plays a significant role in the relationship between immune markers and disease progression. These findings emphasize the critical role that peripheral immune dysregulation plays in amyotrophic lateral sclerosis progression by mediating peripheral nerve injury, particularly in the early stages of the disease. This study highlights the importance of the peripheral nervous system in the early stages of amyotrophic lateral sclerosis and provides new insights into disease mechanisms and potential therapeutic targets.

## Graphical abstract

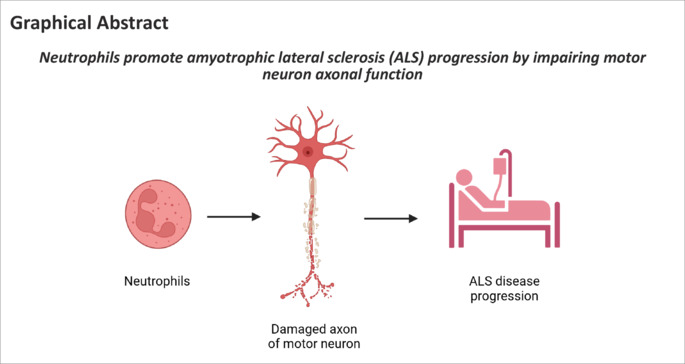

## Introduction

Amyotrophic lateral sclerosis (ALS) is a fatal neurodegenerative disease characterized by progressive loss of upper motor neurons (MNs) and lower MNs (LMNs), leading to increasing muscle weakness that eventually results in death due to respiratory failure (Ilieva et al., 2023). There is currently no cure for ALS, and the goal of current therapies is to prolong survival and enhance quality of life. The average life expectancy for patients with ALS is 3–5 years (Masrori and Van Damme, 2020). The large degree of variation in clinical phenotypes and rates of ALS development among patients (Feldman et al., 2022) make it difficult to investigate the underlying mechanisms and create efficient therapies.

It was previously widely recognized that motor neuron degeneration begins in the motor cortex and progressively descends to the neuromuscular junction in ALS. However, there is mounting evidence that denervation of the neuromuscular junction and axonal degeneration of terminal-type MNs are early steps in the ALS pathogenic cascade. MN axonal dysfunction precedes ALS symptoms in various animal models of the disease (Riva et al., 2014; Verma et al., 2022). We previously showed that the most effective way to measure LMN axonal damage in ALS is to measure the amplitude of the compound muscle action potential (CMAP) (Yu et al., 2021). Furthermore, poor LMN axonal function is linked to poor prognosis and rapid disease progression in patients within 12 months of ALS onset. Serum neurofilament protein light chain is the most promising potential biomarker for ALS. Moreover, we found that increased serum neurofilament protein light chain levels correlate with decreased CMAP amplitude, which may reflect the severity of LMN axonal degeneration (Zhang et al., 2022a). However, the cause of peripheral nerve axonal damage in ALS remains unclear.

In recent years, numerous studies have found that peripheral immune dysregulation is a main contributing factor to ALS pathogenesis. Nardo et al. (2013) observed that 129SvSOD1 mice with faster disease progression exhibited significantly more peripheral nerve axonal damage at disease onset than C57SOD1 mice with slower disease progression. Transcriptome analysis of these two mouse models revealed that the primary differences were related to the immune response. The peripheral immune system comprises both the innate and the adaptive immune systems. The former is dominated by neutrophils, while the latter is dominated by lymphocytes. Different immune cells have different roles in ALS; T regulatory cells seem to be protective (Yazdani et al., 2022), while neutrophils, natural killer (NK) cells, and CD8^+^ T cells may be harmful (Murdock et al., 2017, 2021a, b; Zhao et al., 2017; Cui et al., 2022; Jiang et al., 2024). There is growing evidence that peripheral immune activation is one of the systemic changes that occurs in ALS and contributes to disease progression (Murdock et al., 2017, 2021a, b; Cui et al., 2022; Yazdani et al., 2022; Yu et al., 2022; Jiang et al., 2024). However, the specific role and mechanisms of peripheral immunity in peripheral nerve damage in ALS are still unclear.

Age, sex, and body mass index (BMI) may all be associated with the link between peripheral immunity and ALS. Previous studies have found that neutrophils disproportionately affect survival in female patients with ALS (Murdock et al., 2021a, 2024), whereas NK cell activity is related to disease progression in an age- and sex-dependent manner (Murdock et al., 2021b). Being overweight is associated with low-grade systemic inflammation (Lackey and Olefsky, 2016), and adipocytes can alter the characteristics of peripheral immune cells (Johnson et al., 2012; Frasca et al., 2016; Purdy and Shatzel, 2021). Furthermore, BMI is now thought to be negatively associated with ALS disease progression (Nakken et al., 2019). Thus, the main confounding variables affecting correlations with ALS include age, sex, and BMI. Understanding how peripheral immunity affects particular patient subgroups is essential for developing predictive models, performing clinical trials, and eventually offering patients tailored immunotherapy.

Hence, in this study we examined the connections between LMN axonal impairment and peripheral immune markers in patients with sporadic ALS with disease onset < 12 months. Then, we grouped patients by age, sex, and BMI to identify potential differences among subgroups. Finally, we conducted mediation analyses to explore the potential mechanisms by which peripheral immunity influences disease progression.

## Methods

### Subjects and clinical characteristics

The Peking University Third Hospital’s Ethics Committee approved this clinical cohort study on September 20, 2017 (approval No. 223-02). We obtained informed consent from each patient or their legal representative. The study complies with the *Declaration of Helsinki*.

**Figure 1:**
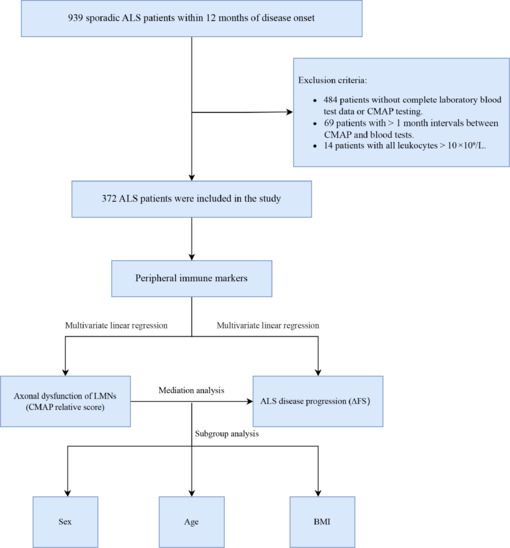
Study flowchart. Initially, 939 patients with ALS with disease duration < 12 months were recruited. After excluding patients with missing laboratory blood tests or CMAP testing data, as well as those who had intervals > 1 month between the two tests or leukocyte count > 10 × 10^9^/L, 372 patients with ALS were included in the analyses. Relationships among peripheral immune markers, LMN axonal dysfunction, and ALS disease progression were analyzed. Subsequently, mediation analysis was conducted to determine whether LMN axonal dysfunction mediates the relationship between peripheral immune markers and disease progression. ALS: Amyotrophic lateral sclerosis; BMI: body mass index; CMAP: compound muscle action potential; LMN: lower motor neuron; ΔFS: disease progression rate.

Between January 2018 and May 2024, 939 individuals with sporadic ALS within 12 months of disease onset who visited Peking University Third Hospital were enrolled in the study. Patients met the El Escorial Revision of the ALS diagnostic criteria (Brooks et al., 2000) including clinically definite, probable, possible, or lab-supported possible ALS. Of these, 484 patients were excluded due to incomplete laboratory blood tests or CMAP tests, 69 were excluded because the interval between the 2 tests exceeded 1 month, and an additional 14 were excluded due to elevated leukocyte counts (> 10 × 10^9^/L) (Yang et al., 2024). Based on these inclusion and exclusion criteria, a total of 372 patients were included in the final analysis (**Figure 1**).

Each patient was assessed at 3-month intervals either during follow-up visits in the clinic or over the telephone. We collected baseline demographic information and clinical data–age, gender, BMI, site of onset, and disease duration–at the patient’s initial visit. Disease severity was assessed using the revised ALS functional rating scale (ALSFRS-R) (van Eijk et al., 2021). Disease progression rate (ΔFS) was calculated as (48 - ALSFRS-R score at the time of diagnosis)/disease duration (months) (Kimura et al., 2006). A decrease in the total ALSFRS-R score indicates deterioration, while a larger ΔFS indicates faster disease progression.

### Blood sample collection and analysis

Peripheral venous blood was collected from patients in the morning after overnight fasting. Blood counts including leukocyte, platelet, lymphocyte, monocyte, eosinophil, basophil, and neutrophil counts, were performed using a fully automated hematology analyzer (Sysmex, Kobe, Japan) at the Laboratory Department of Peking University Third Hospital.

Based on the blood count results, we calculated four ratios, including NLR (neutrophils/lymphocytes), PLR (platelets/lymphocytes), SII (neutrophils × platelets/lymphocytes), and LMR (lymphocytes/monocytes). Neutrophil counts, monocyte counts, NLR, PLR, and SII are generally considered to represent innate immunity, whereas lymphocyte counts and LMR represent adaptive immunity.

### Compound muscle action potential

The CMAP amplitudes of the median, ulnar, tibial, and peroneal nerves in patients with ALS were recorded using a Keypoint four-channel electromyograph evoked potentiometer (Medtronic, Minneapolis, MN, USA). Given the variation in CMAP amplitudes across different nerves and age groups, we followed criteria established by the Neurophysiology Laboratory of Peking University Third Hospital for evaluation. The following scores were assigned according to the degree of functional decline: 0 for normal, 10 for mild reduction, 20 for moderate reduction, and 30 for severe reduction. Then, the sum of the CMAP amplitude scores for the eight nerves (0–240) was calculated to assess overall axonal damage in patients with ALS. We calculated relative axonal damage scores (relative score = absolute score/number of nerves tested; 0–30) for patients who had fewer than eight nerves evaluated. Further information regarding the scoring system is available in our previous study (Zhang et al., 2022a).

### Statistical analysis

The patients were divided into three groups based on CMAP relative scores: the first group included patients in the first quartile, the second group combined the second and third quartiles, and the third group comprised the fourth quartile. Baseline characteristics were compared among the three groups. Interquartile range medians are used to report continuous variables, while percentage frequencies are used to report categorical variables. The Kruskal–Wallis test was employed to compare differences among the groups. Differences between peripheral immune markers were assessed using the Kruskal–Wallis test and *post hoc* Dunn’s test for patients in different CMAP relative score groups. The results are displayed using violin plots. Spearman correlation analysis was applied to examine the relationships between CMAP relative scores and clinical outcomes.

We used linear regression to analyze correlations among peripheral immune cells and their derivatives and disease severity (ALSFRS-R), disease progression (ΔFS), and LMN axonal dysfunction (CMAP relative score). Peripheral immune markers were log-transformed before fitting the model. After adjusting for potential confounders (age, sex, BMI, disease duration, and site of onset), a multiple linear regression model was built to determine if peripheral immunity markers were related to ALSFRS-R, ΔFS, or CMAP relative score. False discovery rate (FDR) correction was applied to the *P* values. Subgroups were analyzed according to age, gender, and BMI. Patients were stratified using an age cut-off of 50 years because this is the average age of menopause in China (Song et al., 2018), and BMI ≥ 25 kg/m^2^ was defined as overweight. To investigate whether there was a stratum impact among various subgroups, interaction analyses were performed for age, sex, and BMI subgroups.

**Figure 2:**
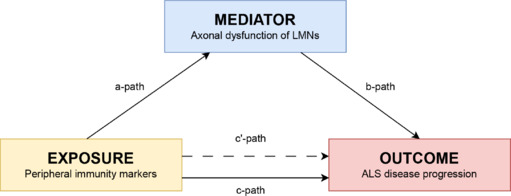
Mediation analysis model paths. This diagram depicts the exploration of whether LMN axonal dysfunction acts as a mediator between exposure (peripheral immune markers) and outcome (ALS disease progression). The entire effect of exposure on outcome is represented by path c; the effect of exposure on mediator is represented by path a; the effect of mediator on outcome is represented by path b; and the direct effect of exposure on outcome is represented by path c’. ALS: Amyotrophic lateral sclerosis; LMN: lower motor neuron.

We explored the potential mediating role of LMN axonal dysfunction between peripheral immunological indicators and disease progression by multivariate regression analysis. For this analysis, the exposure was peripheral immune markers, the outcome was ALS disease progression, and the mediator was LMN axonal dysfunction (**Figure 2**). All models were adjusted for confounders, including age, sex, BMI, disease duration, and site of onset.

*P* < 0.05 was considered statistically significant. R (version 4.2.1; R Foundation for Statistical Computing, Vienna, Austria) and GraphPad Prism (version 9.0.0 for Windows, GraphPad Software, Boston, MA, USA, www.graphpad.com) were used for the statistical analyses.

## Results

### Demographic and clinical characteristics

In total, 939 patients with sporadic ALS and disease onset within ≤ 12 months were initially included in the study. After excluding 484 patients due to incomplete laboratory blood tests or CMAP tests, 69 patients whose testing interval exceeded 1 month, and 14 patients with elevated leukocyte counts (> 10 × 10^9^/L), 372 patients were ultimately included in the analysis (**Figure 1**). Of these, 240 (64.52%) were men. The median age at diagnosis was 54 years, the median disease duration was 250 days, and the median ALSFRS-R score was 44. The patients were divided into three groups based on their CMAP relative scores: 88 (23.66%) in group 1, 155 (41.67%) in group 2, and 129 (36.68%) in group 3. The baseline characteristics of each group are shown in **Table 1**.

**Table 1:** Baseline characteristics according to CMAP relative score

Characteristics	Total	CMAP relative score			
		The first	The second	The third	*P* value
*n*	372	129	155	88	
Sex, *n* (%)					0.805
Male	240 (64.52)	85 (65.89)	97 (62.58)	58 (65.91)	
Female	132 (35.48)	44 (34.11)	58 (37.42)	30 (34.09)	
Age, yr	54.00 (45.00, 61.00)	52.00 (46.00, 59.00)	56.00 (45.00, 62.50)	55.00 (43.00, 63.00)	0.255
BMI, kg/m^2^ (median, IQR)	23.70 (21.95, 25.83)	23.81 (22.04, 25.93)	23.66 (21.80, 26.22)	23.56 (21.62, 25.50)	0.669
ALSFRS-R (median, IQR)	44.00 (40.75, 45.00)	45.00 (42.00, 46.00)	44.00 (41.00, 45.00)	40.00 (33.00, 42.00)	< 0.001***
Disease duration, d (median, IQR)	250 (191, 304)	243.00 (174, 301)	233.00 (185, 296)	270.00 (230, 315)	0.001***
CMAP relative score (median, IQR)	5.00 (2.50, 8.75)	1.25 (0.00, 2.50)	5.00 (3.75, 7.50)	13.12 (11.25, 16.25)	< 0.001***
Site of onsite, *n* (%)					< 0.001***
Bulbar	69 (18.55)	47 (36.43)	17 (10.97)	5 (5.68)	
Cervical	188 (50.54)	43 (33.33)	86 (55.48)	59 (67.05)	
Lumbar	94 (25.27)	31 (24.03)	43 (27.74)	20 (22.73)	

The first group included the first quartile of the CMAP relative score, the second group included the second and third quartiles of the CMAP relative score, and the third group included the fourth quartile of the CMAP relative score. For continuous variables, data are displayed as medians (quartiles), and for categorical variables, as numbers (percentages). ***P < 0.001 (Kruskal-Wallis test). ALSFRS-R: Revised amyotrophic lateral sclerosis functional rating scale; BMI: body mass index; CMAP: compound muscle action potential; IQR: interquartile range.

### Association of peripheral immune markers with disease progression in amyotrophic lateral sclerosis

First, we looked into the relationship between ALS functional status and peripheral immunological markers. The results from the multivariate linear regression showed that ALSFRS-R scores were negatively correlated with leukocytes (*P* = 0.001), neutrophils (*P* < 0.001), NLR (*P* = 0.037), and SII (*P* = 0.007), after adjusting for age, sex, BMI, disease duration, and site of onset. Following FDR correction, these relationships were still valid for leukocytes (*P* = 0.007), neutrophils (*P* = 0.002), and SII (*P* = 0.025) (**Additional Table 1**). These findings suggest that poorer functional status in ALS patients is strongly associated with increased peripheral immune markers (especially leukocytes, neutrophils, and SII).

**Additional Table 1:** Univariate and multivariate linear regression analyses of peripheral immune markers and disease severity (ALSFRS-R scores) in ALS patients within 12 months of disease onset

Peripheral immune marker	Unajusted		Adjusted for age, sex, BMI, disease duration and site of onsite		
	
	β (95% CI)	*P* value	*P* (95% CI)	*P* value	*P* FDR
All leukocytes	-2.294 (-4.425, -0.163)	0.035*	-3.471 (-5.568,-1.375)	0.001**	0.007**
Platelets	-1.282 (-3.202, 0.639)	0.190	-1.698 (-3.634, 0.238)	0.085	0.188
Monocytes	-0.633 (-2.307, 1.041)	0.457	-0.88 (-2.568, 0.808)	0.306	0.421
Lymphocytes	-0.521 (-2.323, 1.28)	0.570	-1.22 (-2.989, 0.55)	0.176	0.323
Basophils	-0.091 (-0.36, 0.178)	0.506	-0.105 (-0.367, 0.158)	0.434	0.531
Eosinophils	0.605 (-0.179, 1.389)	0.130	0.471 (-0.315, 1.257)	0.239	0.376
Neutrophils	-2.258 (-3.89, -0.625)	0.007**	-3.072 (-4.678,-1.466)	<0.001***	0.002**
NLR	-1.326 (-2.726, 0.074)	0.063	-1.468 (-2.851, -0.086)	0.037*	0.103
PLR	-0.469 (-2.06, 1.121)	0.562	-0.151 (-1.713, 1.411)	0.850	0.850
SII	-1.377 (-2.533, -0.222)	0.020*	-1.555 (-2.679, -0.432)	0.007**	0.025*
LMR	0.145 (-1.346, 1.636)	0.849	-0.185 (-1.709, 1.339)	0.812	0.850

*P < 0.05, **P < 0.01, ***P < 0.001. 95% CI: 95% confidence interval; ALS: amyotrophic lateral sclerosis; ALSFRS-R: revised amyotrophic lateral sclerosis functional rating scale; BMI: body mass index; FDR: false discovery rate; LMR: lymphocyte-to-monocyte ratio; NLR: neutrophil-to-lymphocyte ratio; PLR: platelet-to-lymphocyte ratio; SII: systemic immune-inflammation index.

Next, we examined the connection between peripheral immune markers and ALS disease progression (ΔFS). According to the multivariate linear regression model, leukocytes (*P* = 0.006), neutrophils (*P* < 0.001), NLR (*P* = 0.026), and SII (*P* = 0.005) were positively correlated with ΔFS. Following FDR correction, these relationships remained significant for leukocytes (*P* = 0.022), neutrophils (*P* = 0.005), and SII (*P* = 0.022) (**Additional Table 2**). These results highlight the positive correlation between higher levels of leukocytes, neutrophils, and SII with accelerated disease progression in ALS patients.

**Additional Table 2:** Univariate and multivariate linear regression analyses of peripheral immune markers and disease progression (AFS) in ALS patients within 12 months of disease onset

Peripheral immune markers	Unadjusted		Adjusted for age, sex, BMI, disease duration and site of onsite		
	
	β (95% CI)	*P* value	*P* (95% CI)	*P* value	*P* FDR
All leukocytes	0.325 (0.060, 0.591)	0.016*	0.376 (0.109, 0.643)	0.006**	0.022*
Platelets	0.193 (-0.047, 0.432)	0.114	0.209 (-0.037, 0.454)	0.095	0.210
Monocytes	0.059 (-0.15, 0.267)	0.582	0.080 (-0.134, 0.294)	0.463	0.566
Lymphocytes	0.107 (-0.118, 0.331)	0.351	0.107 (-0.117, 0.332)	0.348	0.546
Basophils	0.006 (-0.028, 0.039)	0.746	0.013 (-0.021, 0.046)	0.452	0.566
Eosinophils	-0.06 (-0.158, 0.038)	0.227	-0.054 (-0.154, 0.045)	0.284	0.521
Neutrophils	0.323 (0.12, 0.526)	0.002**	0.368 (0.164, 0.572)	<0.001***	0.005**
NLR	0.170 (-0.005, 0.345)	0.056	0.200 (0.025, 0.375)	0.026*	0.070
PLR	0.049 (-0.150, 0.247)	0.630	0.052 (-0.146, 0.249)	0.609	0.670
SII	0.187 (0.043, 0.331)	0.011*	0.204 (0.062, 0.346)	0.005**	0.022*
LMR	0.027 (-0.159, 0.213)	0.779	0.014 (-0.179, 0.207)	0.885	0.885

*P < 0.05, **P < 0.01, ***P < 0.001. 95% CI: 95% confidence interval; ALS: amyotrophic lateral sclerosis; BMI: body mass index; FDR: false discovery rate; LMR: lymphocyte-to-monocyte ratio; NLR: neutrophil-to-lymphocyte ratio; PLR: platelet-to-lymphocyte ratio; SII: systemic immune-inflammation index; AFS: disease progression rate.

No significant correlations were found between platelets, monocytes, lymphocytes, basophils, eosinophils, and ALSFRS-R or ΔFS. This shows that not all peripheral immune markers are associated with ALS progression.

### Association between compound muscle action potential relative score and baseline clinical indicators

**Figure 3:**
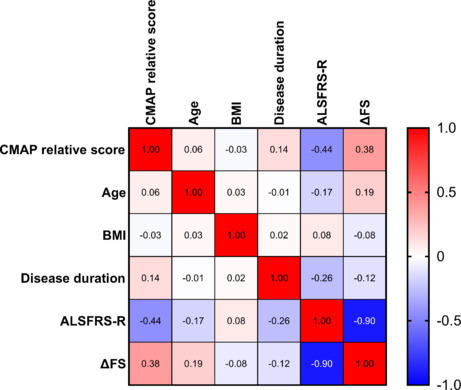
Correlation of CMAP relative score with clinical parameters in patients with ALS. Red indicates positive correlations, whereas blue indicates negative correlations. More intense colors indicate more significant correlations based on the absolute value of the coefficient. ALS: Amyotrophic lateral sclerosis; ALSFRS-R: revised amyotrophic lateral sclerosis functional rating scale; BMI: body mass index; CMAP: compound muscle action potential; ΔFS: disease progression rate.

In this section, we further explored the relationship between LMN axonal function and clinical features of ALS. We analyzed the correlations between CMAP relative score and other clinical indicators of ALS, such as age, BMI, disease duration, ALSFRS-R, and ΔFS. CMAP relative score was positively correlated with disease duration (*ρ* = 0.138, *P* = 0.008) and ΔFS (*ρ* = 0.382, *P* < 0.001). In contrast, CMAP relative score was negatively correlated with ALSFRS-R (*ρ* = –0.440, *P* < 0.001). There were no statistically significant relationships between age or BMI and CMAP relative score. These findings highlight an association between LMN axonal damage and ALS disease duration, functional status, and progression (**Figure 3**). This emphasizes the importance of CMAP as a potential marker of disease progression in ALS.

### Associations between peripheral immune markers and lower motor neuron axonal dysfunction

**Figure 4:**
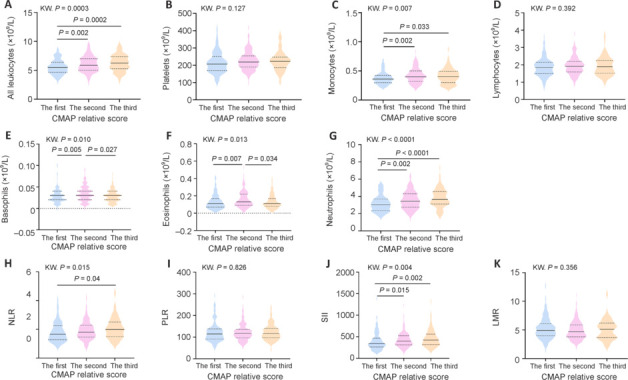
Comparison of peripheral immune markers (all leukocytes, platelets, monocytes, lymphocytes, basophils, eosinophils, neutrophils, NLR, PLR, SII, and LMR) among different groups based on CMAP relative score. All leukocyte count (A), monocyte count (C), basophil count (E), eosinophil count (F) and neutrophil count (G), as well as NLR (H) and SII (J), showed statistically significant differences among the different CMAP relative score groups. In particular, all leukocyte count (A), monocyte count (C), neutrophil count (G), NLR (H), and SII (J) increased as the CMAP relative score increased. However, there were no statistically significant differences in platelets (B), lymphocytes (D), PLR (I), or LMR (K) among the different CMAP relative score groups. The frequency distribution of data is displayed as violin plots, in which the width of the sections indicates the proportion of a given value (wider sections indicate a larger proportion). The medians are shown as solid lines, and the interquartile ranges are shown as dotted lines. CMAP relative scores were grouped according to quartiles, with the first group representing the first quartile, the second group representing the second and third quartiles, and the third group representing the fourth quartile. Groups were compared by Kruskal–Wallis (KW) test and *post hoc* Dunn’s test. CMAP: Compound muscle action potential; LMR: lymphocyte-to-monocyte ratio; NLR: neutrophil-to-lymphocyte ratio; PLR: platelet-to-lymphocyte ratio; SII: systemic immune-inflammation index.

To explore the relationship between peripheral immune markers and LMN axonal dysfunction in ALS, we observed significant differences in peripheral immune markers across the CMAP relative score groups. Specifically, total leukocyte (*P* < 0.001), monocyte (*P* = 0.033), basophil (*P* = 0.010), eosinophil (*P* = 0.013), and neutrophil (*P* < 0.001) counts, as well as NLR (*P* = 0.015) and SII (*P* = 0.004), varied significantly among the groups. Notably, total leukocyte, monocyte, and neutrophil counts and NLR and SII increased with increasing CMAP relative scores. These differences remained statistically significant even after applying multiple comparison corrections (**Figure 4**). The median (interquartile range) of the peripheral immune cell counts and four derived ratios are shown in **Additional Table 3**.

**Additional Table 3:** Peripheral immune markers across different CMAP relative score groups in ALS patients within 12 months of disease onset

Peripheral immune marker	Total	CMAP relative score			
		The first	The second	The third	*P* value
All leukocytes (×10^9^ cells/L)	5.78 (4.91, 6.93)	5.46 (4.67, 6.40)	5.86 (5.02, 7.01)	6.25 (5.30, 7.34)	<0.001***
Platelets (×10^9^ cells/L)	216.00 (180.75, 252.25)	207.00 (170.00, 250.00)	219.00 (190.00, 253.50)	222.50 (185.75, 247.00)	0.127
Monocytes (×10^9^ cells/L)	0.39 (0.31, 0.47)	0.36 (0.30, 0.42)	0.40 (0.32, 0.50)	0.41 (0.31, 0.49)	0.007**
Lymphocytes (×10^9^ cells/L)	1.89 (1.53, 2.19)	1.84 (1.49, 2.14)	1.92 (1.59, 2.18)	1.90 (1.51, 2.25)	0.392
Basophils (×10^9^ cells/L)	0.03 (0.02, 0.04)	0.03 (0.02, 0.04)	0.03 (0.02, 0.04)	0.03 (0.02, 0.04)	0.010**
Eosinophils (×10^9^ cells/L)	0.12 (0.08, 0.19)	0.11 (0.07, 0.17)	0.13 (0.09, 0.22)	0.11 (0.08, 0.17)	0.013*
Neutrophils (×10^9^ cells/L))	3.33 (2.73, 4.17)	3.04 (2.37, 3.67)	3.43 (2.74, 4.28)	3.63 (3.12, 4.54)	<0.001***
NLR	1.81 (1.43, 2.30)	1.66 (1.29, 2.25)	1.81 (1.48, 2.27)	1.98 (1.51, 2.47)	0.015*
PLR	115.87 (95.05, 137.85)	114.50 (91.30, 137.21)	116.67 (96.83, 135.28)	116.24 (97.11, 140.51)	0.826
SII	381.66 (296.43, 523.03)	339.06 (263.90, 468.83)	398.02 (312.23, 522.41)	423.17 (321.37, 558.51)	0.004**
LMR	4.83 (3.82, 5.97)	4.93 (4.00, 6.07)	4.68 (3.80, 5.87)	5.10 (3.67, 6.15)	0.356

The first group included the first quartile of the CMAP relative score, the second group included the second and third quartiles of the CMAP relative score, and the third group included the fourth quartile of the CMAP relative score. The data are presented with the median (interquartile range). CMAP relative scores are grouped according to quartile spacing, with the first representing the first quartile spacing group, the second representing the second and third quartile spacing groups, and the third representing the fourth quartile spacing group. The Kruskal-Wallis test was employed to compare differences among the groups. *P < 0.05, **P < 0.01, ***P < 0.001. ALS: Amyotrophic lateral sclerosis; CMAP: compound muscle action potential; IQR: interquartile range; LMR: lymphocyte-to-monocyte ratio; NLR: neutrophil-to-lymphocyte ratio; PLR: platelet-to-lymphocyte ratio; SII: systemic immune-inflammation index.

To investigate the connection between peripheral immune markers and CMAP relative score, linear regression analysis was performed. In the univariate analysis, increased leukocyte (*P* = 0.008) and neutrophil (*P* < 0.001) counts were substantially linked to more severe LMN axonal dysfunction. Higher innate immunity ratios, such as NLR (*P* = 0.007) and SII (*P* = 0.004), were also significantly associated with the degree of LMN axonal dysfunction. These associations persisted in the multivariate linear regression after adjusting for covariates, including age, sex, BMI, disease duration, and site of onset. Specifically, higher leukocyte counts (*P* = 0.005), neutrophil counts (*P* < 0.001), NLR (*P* = 0.036), and SII (*P* = 0.020) were confirmed to be linked with more severe LMN axonal dysfunction in ALS. Additionally, the correlations between leukocyte counts (*P* = 0.029) and neutrophil counts (*P* = 0.008) and LMN axonal dysfunction remained significant after FDR correction. No significant correlations were found between platelets, monocytes, lymphocytes, basophils, or eosinophils and LMN axonal damage in either the univariate or multivariate models (**Table 2**).

**Table 2:** Univariate and multivariate linear regression analyses of peripheral immune markers and LMN axonal dysfunction (CMAP relative score) in ALS patients within 12 months of disease onset

Peripheral immune marker	Unadjusted			Adjusted for age, sex, BMI, disease duration and site of onsite		
		
	β (95% CI)	*P* value		β (95% CI)	*P* value	P FDR
All leukocytes	3.056 (0.8, 5.312)	0.008**		3.097 (0.929, 5.264)	0.005**	0.029*
Platelets	1.064 (–0.978, 3.106)	0.306		1.070 (–0.93, 3.069)	0.293	0.518
Monocytes	0.798 (–0.98, 2.575)	0.378		0.893 (–0.846, 2.631)	0.313	0.518
Lymphocytes	0.446 (–1.468, 2.36)	0.647		0.905 (-0.919, 2.73)	0.330	0.518
Basophils	0.053 (–0.233, 0.339)	0.716		–0.062 (–0.333, 0.209)	0.653	0.898
Eosinophils	–0.112 (–0.947, 0.724)	0.793		0.049 (–0.762, 0.86)	0.905	0.989
Neutrophils	3.178 (1.457, 4.899)	< 0.001***		2.877 (1.217, 4.536)	< 0.001***	0.008**
NLR	2.041 (0.562, 3.521)	0.007**		1.520 (0.096, 2.944)	0.036*	0.100
PLR	0.379 (–1.311, 2.069)	0.659		–0.011 (–1.62, 1.598)	0.989	0.989
SII	1.788 (0.565, 3.012)	0.004**		1.376 (0.216, 2.536)	0.020*	0.074
LMR	–0.326 (–1.91, 1.257)	0.685		–0.057 (–1.628, 1.513)	0.943	0.989

*P < 0.05, **P < 0.01, ***P < 0.001. 95% CI: 95% Confidence interval; ALS: amyotrophic lateral sclerosis; BMI: body mass index; CMAP: compound muscle action potential; FDR: false discovery rate; LMN: lower motor neuron; LMR: lymphocyte-to-monocyte ratio; NLR: neutrophil-to-lymphocyte ratio; PLR: platelet-to-lymphocyte ratio; SII: systemic immune-inflammation index.

These results show that LMN axonal dysfunction in ALS is strongly correlated with peripheral immune markers, specifically leukocyte and neutrophil counts. This emphasizes the potential role of peripheral immune markers in ALS progression.

### Stratified analysis

**Figure 5:**
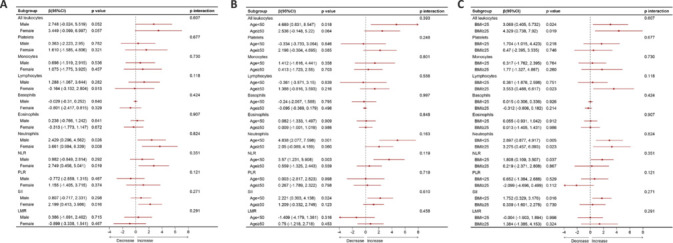
Forest plot of associations between peripheral immune markers and LMN axonal dysfunction (CMAP relative score) stratified by sex, age, and BMI. (A) Sex-stratified analysis. (B) Age-stratified analysis. (C) BMI-stratified analysis. 95% CI: 95% Confidence interval; BMI: body mass index; CMAP: compound muscle action potential; LMN: lower motor neuron; LMR: lymphocyte-to-monocyte ratio; NLR: neutrophil-to-lymphocyte ratio; PLR: platelet-to-lymphocyte ratio; SII: systemic immune-inflammation index.

To further explore potential differences between peripheral immune markers and LMN axonal dysfunction, we performed subgroup analyses grouped by sex, age, and BMI. Although the interaction analysis did not yield significant results, the stratified analyses revealed some noteworthy patterns. The relationships between NLR and SII and LMN axonal dysfunction were more significant in females than in males (NLR: *P* = 0.019; SII: *P* = 0.016). Age-stratified analysis showed that patients younger than 50 years exhibited a stronger relationship between elevated leukocytes (*P* = 0.018), neutrophils (*P* = 0.001), NLR (*P* = 0.003), and SII (*P* = 0.024) and more severe LMN axonal dysfunction than patients older than 50 years. However, there was no difference in the correlation of peripheral immune markers with LMN axonal dysfunction between patients with a BMI over 25 kg/m^2^ or under 25 kg/m^2^ (**Figure 5**). Stratified analyses show that the association between peripheral immune markers and LMN axonal dysfunction may be influenced by sex and age.

### Mediation analysis

To investigate the connections between peripheral immune indicators and disease progression, we constructed a mediation model with LMN axonal dysfunction as the mediator after a significant correlation was confirmed by multivariate regression. Confounding factors were adjusted for in all mediation analyses, including age, sex, BMI, disease duration, and site of onset. The mediation model was statistically significant for leukocytes, neutrophils, NLR, and SII as independent variables. LMN axonal dysfunction accounted for 57.4% of the effect on disease progression in leukocytes, 53.9% in neutrophils, 53.3% in NLR, and 47.0% in SII (**Table 3**). These findings suggest that LMN axonal dysfunction plays an important role in mediating the impact of peripheral immune markers on ALS disease progression.

**Table 3:** Mediation analysis of the effect of peripheral immune markers on disease progression through LMN axonal dysfunction (CMAP relative score)

	All leukocytes			Neutrophils			NLR			SII	
						
	β (95% CI)	*P*		β (95% CI)	*P*		β (95% CI)	*P*		β (95% CI)	*P*
ACME	0.216 (0.046, 0.400)	0.010**		0.192 (0.066, 0.340)	0.002**		0.106 (0.009, 0.200)	0.028*		0.096 (0.016, 0.180)	0.024*
ADE	0.160 (–0.095, 0.390)	0.194		0.170 (0.001, 0.340)	0.050*		0.093 (–0.042, 0.220)	0.182		0.108 (–0.014, 0.230)	0.068
Total effect	0.376 (0.097, 0.650)	0.008**		0.368 (0.167, 0.570)	0.002**		0.200 (0.033, 0.360)	0.050*		0.204 (0.067, 0.340)	< 0.001***
Prop mediated	0.574 (0.160, 1.530)	0.018*		0.539 (0.250, 0.990)	0.004**		0.533 (0.047, 1.640)	0.044*		0.470 (0.121, 1.150)	0.024*

All models were adjusted for age, sex, body mass index, disease duration, and site of onset. *P < 0.05, **P < 0.01, ***P < 0.001. 95% CI: 95% Confidence interval; ACEM: average causal mediation effects; ADE: average direct effects; CMAP: compound muscle action potential; LMNs: lower motor neurons; NLR: neutrophil-to-lymphocyte ratio; Prop mediated: conceptually average causal mediation effects/total effect; SII: systemic immune-inflammation index.

## Discussion

In this study, we investigated for the first time the role of peripheral immune-mediated nerve injury in ALS onset. While ALS is primarily considered to be a disease of the central nervous system (Giacomelli et al., 2022), our findings, along with those of prior studies, indicate that patients with ALS who have more severe peripheral nerve damage experience faster disease progression (Gaiani et al., 2017; Sugimoto et al., 2020; Yu et al., 2021). Even though new evidence indicates that the immune system is crucial to ALS onset, is the mechanisms underlying this effect remain unknown. Using mediation analysis, our study is the first to elucidate the relationship between peripheral immunity and peripheral nerve injury.

This study included 372 patients with sporadic ALS, all within 12 months of disease onset. We identified an association between leukocyte count, neutrophil count, NLR, and SII and LMN axonal dysfunction. Subgroup analyses revealed that this association was particularly significant in female patients and those younger than 50 years. Subsequent mediation analysis identified LMN axonal impairment as a mediating component in the relationship between illness progression and peripheral immunological markers.

The peripheral immune system is made up of the innate and adaptive immune systems. Routine hematology testing is the simplest and most common method used to assess peripheral immunity, including leukocytes, platelets, monocytes, lymphocytes, eosinophils, basophils, and neutrophils. Furthermore, there has been a growing focus on a number of immune ratios, such as NLR, PLR, LMR, and SII. It is believed that innate immunity is represented by neutrophils, monocytes, NLR, and SII, while adaptive immunity is represented by lymphocytes and the LMR (Jensen et al., 2021; Zhang et al., 2022b).

As the predominant leukocytes in the peripheral circulation, the role of neutrophils in ALS has been demonstrated on several levels. Compared with healthy controls, patients ALS have higher neutrophil counts (Murdock et al., 2017). In addition, neutrophil counts increase over time and are significantly correlated with lower ALSFRS-R scores (Murdock et al., 2017; Jiang et al., 2024). Moreover, elevated neutrophil counts in early ALS are associated with shorter survival (Murdock et al., 2021a; Cotet et al., 2023; Grassano et al., 2023). Furthermore, our study using data from the UK Biobank showed that higher neutrophil counts and NLR and SII are associated with a higher risk of ALS (Cao et al., 2023). Finally, a recent study reported that neutrophils are linked to cognitive impairment in sporadic ALS (Yang et al., 2024). The relationship between neutrophils and ALS is also supported by animal experiments. A study showed that neutrophils infiltrate around motor nerve axons in SOD1G93A rats. Treating these rats with the tyrosine kinase inhibitor masertinib reduced neutrophil infiltration, which in turn improved axonal damage and demyelination (Trias et al., 2018), suggesting that neutrophils may contribute to peripheral neurodegeneration in ALS. NLR and SII are commonly used to assess the balance between innate and adaptive immunity (Zahorec, 2021; Xie et al., 2022). An increased NLR is a reliable indicator of poor prognosis and rapid disease progression in patients with ALS (Choi et al., 2020; Leone et al., 2022; Wei et al., 2022), while an elevated SII is associated with a higher probability of incident ALS (Cao et al., 2023). Our study is the first to show a connection between increased neutrophil counts, NLR, and SII and LMN axonal impairment in the early phases of ALS development.

Our results showed that LMN axonal impairment and peripheral immune markers were most strongly associated with female patients with ALS under 50 years of age. This suggests that the immune mechanisms associated with axonal damage in ALS may vary by population subgroup. Future therapeutic approaches and clinical trial designs should take this into account. Previous research has identified sex-based differences in peripheral nerve dysfunction and immunology in ALS. For example, one study found that axonal regeneration varies between male and female SOD1G37R mice (Martineau et al., 2020). Additionally, females are more affected than males by the effects of neutrophils, NK cells, and immunological profiles during the course of ALS (Murdock et al., 2021a, b, 2024), which aligns with our findings. However, the mechanisms underlying these differences in ALS remain unclear. Potential factors include hormonal effects on immune cell function or genetic variations (Jacob et al., 2024). For example, testosterone reduces neutrophil bioactivity (Marin et al., 2010), while progesterone and estradiol delay neutrophil apoptosis and enhance neutrophil function (Molloy et al., 2003). Analysis of blood from ALS patients revealed that men and women express “switch genes” differently. Switch genes are significantly linked to metabolic pathways in men, while they are linked to inflammatory conditions in women (Santiago et al., 2021). Additionally, consistent with our earlier study (Cao et al., 2023), we found that that the connection between neutrophils and LMN axonal dysfunction was age-dependent. This may be because of the decrease in neutrophil phagocytosis and degranulation that occurs with aging (Braga et al., 1998; Schröder and Rink, 2003). However, a recent study found a stronger association between neutrophils and ALS disease progression in older adults (Murdock et al., 2024). The median age of the patients included in that study was 65 years, which could account for the apparent lack of variance among younger patients.

Neutrophils function through phagocytosis, degranulation, and the secretion of neutrophil extracellular traps (Cao and Fan, 2023). However, the relationship between neutrophils and peripheral nerve axonal injury in ALS remains unclear. There are several potential mechanisms. Firstly, because of their small size compared with that of MNs, neutrophils may not directly engage in MN axon phagocytosis in ALS. In addition, neutrophils release a variety of enzymes, such as matrix metalloproteinases, myeloperoxidase, and neutrophil elastase (Amulic et al., 2012; Sheshachalam et al., 2014). Notably, some studies have reported elevated serum matrix metalloproteinase-9 levels in patients with ALS prior to onset of muscular dystrophy and peripheral neurodegeneration (Demestre et al., 2005). Additionally, matrix metalloproteinase-9 downregulation in the lumbar neurons of a mouse model of ALS postponed axonal damage and improved MN survival (Spiller et al., 2019). Moreover, myeloperoxidase induces apoptosis in necrotic cells by generating the oxidant hypothiocyanous acid and further leads to axonal degeneration in ALS (Ito et al., 2016; Bozonet et al., 2023). Neutrophil extracellular traps are also formed by neutrophils near the neuromuscular junction in ALS mice and patients with ALS, which may contribute to axonal damage (Trias et al., 2018). In conclusion, neutrophils may contribute to peripheral nerve axonal injury in ALS through degranulation and the formation of neutrophil extracellular traps, which may drive disease progression.

Adaptive immunity, which is mediated by lymphocytes, has a complicated function in ALS. CD4^+^ T cells prolong survival in ALS mice and patients with ALS (Beers et al., 2011; Henkel et al., 2013; Sheean et al., 2018). In contrast, compared with healthy controls, patients with ALS exhibit elevated NK cell counts (Garofalo et al., 2020). We did not observe any correlation between lymphocytes and axonal damage or disease progression in our study. This may be because we did not perform a detailed phenotypic analysis of the entire lymphocyte population. Additionally, other peripheral immune indicators, including monocytes, eosinophils, basophils, and platelets, did not significantly correlate with axonal injury or disease progression. Previous studies have noted changes in the proportion of monocytes in patients with ALS (Murdock et al., 2017); however, only CD16^–^ monocytes were linked to disease progression (Murdock et al., 2016). Moreover, circulating monocytes in ALS tend to differentiate into a pro-inflammatory phenotype that produces more inflammatory factors (Du et al., 2020). However, in our study we did not specifically characterize monocytes. Eosinophils and basophils primarily play regulatory roles in parasitic infections and allergic reactions, and studies on their involvement in ALS are limited. According to one study, patients with ALS have higher eosinophil counts, which are negatively correlated with disease progression (Yang et al., 2023). This apparent discrepancy with our study may be because Yang et al.’s study included a smaller sample size (59 participants), had low statistical power, and did not specify the disease stage of the patients who were included.

Our study included patients within 12 months of disease onset. Given that the median survival period for ALS patients is 2 to 4 years, 12 months is regarded as a relatively early stage of the disease. Patients within this timeframe are often selected for clinical trials. For example, the therapeutic effect of edaravone was found to be significant in patients within 2 years of ALS onset (Writing Group and Edaravone (MCI-186) ALS 19 Study Group, 2017). Similarly, the efficacy of methylcobalamin in ALS was confirmed only through a post hoc analysis of patients diagnosed within 12 months of symptom onset (Kaji et al., 2019; Oki et al., 2022). Mitsumoto et al. (2014) demonstrated that LMNs decay exponentially in ALS, with their number halving within 1 year after symptom onset. If treatment is delayed until later in disease progression, very few LMNs remain to support a large number of muscle fibers. These results emphasize how crucial early intervention is for patients with ALS. Our study elucidates the pathophysiological changes that occur in the early stages of disease, providing a foundation for further exploration of therapeutic targets.

This study had some limitations. First, the relatively limited sample size may have led to selection bias, and thus a large multi-center sample study is needed to validate and support our findings. Second, although multiple covariates were adjusted for in the analysis, longitudinal studies are needed to further clarify causality. Finally, this study mainly focused on the clinical correlation between peripheral immunity and LMN axonal dysfunction, and the specific molecular mechanisms should be explored in more depth.

In conclusion, we showed that, in patients with early-onset ALS, neutrophils and neutrophil-derived markers like NLR and SII are linked to LMN axonal dysfunction. Notably, this relationship was more pronounced in younger female patients. Furthermore, peripheral immune indicators and illness progression were found to be mediated by LMN axonal impairment. Our findings elucidate the potential mechanisms by which peripheral immunity influences ALS. Understanding these molecular foundations could help develop therapeutic targets and biomarkers.

## Additional files:

***Additional Table 1:*** *Univariate and multivariate linear regression analyses of peripheral immune markers and disease severity (ALSFRS-R scores) in ALS patients within 12 months of disease onset.*

***Additional Table 2:*** *Univariate and multivariate linear regression analyses of peripheral immune markers and disease progression (ΔFS) in ALS patients within 12 months of disease onset.*

***Additional Table 3:*** *Peripheral immune markers across different CMAP relative score groups in ALS patients within 12 months of disease onset.*

## Data Availability

*All data relevant to the study are included in the article or uploaded as Additional files*.
